# Charge Trapping and Emission during Bias Temperature Stressing of Schottky Gate GaN-on-Silicon HEMT Structures Targeting RF/mm Wave Power Amplifiers

**DOI:** 10.3390/mi15080951

**Published:** 2024-07-24

**Authors:** Barry O’Sullivan, Aarti Rathi, Alireza Alian, Sachin Yadav, Hao Yu, Arturo Sibaja-Hernandez, Uthayasankaran Peralagu, Bertrand Parvais, Adrian Chasin, Nadine Collaert

**Affiliations:** 1 IMEC, Kapeldreef 75, 3001 Leuven, Belgium; 2 Faculty of Engineering, Vrije Universiteit Brussel, 1050 Ixelles, Belgium

**Keywords:** GaN on Si, Schottky gate HEMT, charge trapping, C-GaN back barrier

## Abstract

For operation as power amplifiers in RF applications, high electron mobility transistor (HEMT) structures are subjected to a range of bias conditions, applied at both the gate and drain terminals, as the device is biased from the OFF- to ON-state conditions. The stability of the device threshold voltage (V_t_) condition is imperative from a circuit-design perspective and is the focus of this study, where stresses in both the ON and OFF states are explored. We see rapid positive threshold voltage increases under negative bias stress and subsequent recovery (i.e., V_t_ reduces), whereas conversely, we see a negative V_t_ shift under positive stress and V_t_ increase during the subsequent relaxation phase. These effects are correlated with the thickness of the GaN layer and ultimately result from the deep carbon-acceptor levels in the C-GaN back barrier incorporated to screen the buffer between the silicon substrate and the epitaxially grown GaN layer. Methods to mitigate this effect are explored, and the consequences are discussed.

## 1. Introduction

The quest for continued enhancements of internet speeds, with the advent of the Internet of Things, artificial intelligence, and virtual reality, requires evolutions in device technologies, and concurrently, device-to-device communications. Research is ongoing into technologies for application in the fifth generation of mobile networks (5G), which can operate at sufficient bandwidth and provide the communication speeds required for these applications. Such devices can operate within the frequency bands labeled as FR3 (~7–25 GHz) and FR2 (24–71 GHz) ranges, which have been assigned for communications. In these frequency ranges, it has been shown that GaN shows superior output power for frequencies up to 100 GHz, above which InP is more promising for the 6G node [1].

However, GaN substrates are produced in sizes up to 6” [2], but given the substrate costs, the intrinsic benefits of GaN, such as the high breakdown and high mobility potential, cannot be commercially exploited in this form. Research efforts are ongoing to integrate GaN on SiC substrates, which have lower costs than GaN substrates and are available in up to a 6” wafer size [3], or GaN on Si, which has been recently reported to have up to 200 mm [1] and 300 mm substrates diameters [4,5]. The scaling of these GaN on silicon technologies results in significant promise for millimetre wave applications, given that it could be integrated into streamlined silicon production facilities. However, given the lattice mismatch between the underlying SiC or Si substrate, the epitaxially grown GaN layers are prone to significant densities of defects [6]. Efforts to mitigate these defect regions’ impact include inserting thick buffer layers and back-barrier layers. While the effect of the defects can be screened by these, in the case of back barriers, AlGaN and C-GaN are frequently applied, which introduce their own challenges, including the lower thermal conductivity of AlGaN, resulting in self-heating challenges [7], whereas the carbon-doping level in p-type C-GaN [8] can interact with, and degrade the device’s operation [9].

During operation in class AB, power-amplifier devices are subjected to varying drain and gate biases (V_d_ and V_g_, respectively), resulting in the application of bias when the device could be biased in the OFF state (negative V_g_), semi-on state (moderate V_g_, moderate V_d_), ON-state (high V_g_, low V_d_), or an intermediate zone between these. The impact of ON-state stress on R_on_ dispersion of Schottky gate structures has been explored [10,11,12] and shown to be related to charge trapping in AlGaN barrier defects, GaN/buffer defects, and AlGaN/SiN surface states.

In this work, we explore the region of low drain and varying gate bias stressing and study the charge trapping and emission characteristics for a range of Schottky gate HEMT structures. We show a correlation with the distance between the 2DEG channel and the C-GaN back barrier on the threshold voltage shifts under both positive (similar to [13]) and negative gate bias stress. Due to the varied bias range applied in RF operation [14], insights into the underlying physical degradation mechanisms are critical and can more readily be learned from DC conditions, such as those performed in this study.

## 2. Materials and Methods

The starting materials were high-resistivity 200 mm Si substrates, onto which epitaxial GaN-based layers were monolithically integrated, as described in [15]. To screen the impact of the buffer layer and its inherent defectivity, a 1 μm carbon-doped GaN back barrier was incorporated underneath the (unintentionally doped) GaN channel. This GaN channel thickness varied between 35 and 300 nm in this study. A thin AlN layer (1 nm) was deposited above the channel, followed by the 15 nm AlGaN barrier layer, which was passivated with a SiN_x_/Al_2_O_3_ stack. The gate region was defined by etching through the passivation layers and partially through the AlGaN barrier to create Schottky gate or HEMT structures. In this case, the final AlGaN thickness under the metal electrode was 8 nm. The polarization charge in the AlGaN/AlN creates a 2D electron gas (2DEG) at the GaN surface, and thus, these devices are in the on state when unbiased. To switch off the device, negative gate bias is applied, which in practice switches off the 2DEG under the gate to limit the source-drain current flow in depletion-mode (D-mode) devices. The dc and RF characteristics of these structures have previously been reported [15] and show that the thinner GaN channel shows improved drain-induced barrier lowering (DIBL), I_off_, and V_t_ roll-off but also shows degraded RF parameters, including cutoff frequency (f_T_), maximum frequency (f_max_), power-added efficiency (PAE), thermal conductance, and charge trapping when biased in the semi-ON state.

**Figure 1:**
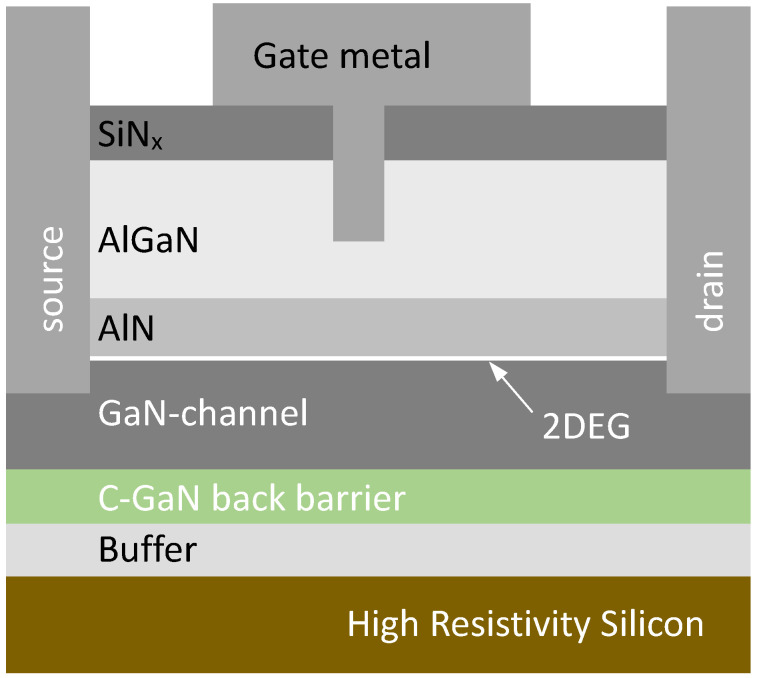
Schematic diagram of HEMT structures as applied in this work and fabricated on 200 mm silicon substrates.

In this study, variations are made in the GaN channel thickness, from 35 to 300 nm, together with the type of back barrier incorporated between the buffer layer and the channel. A schematic diagram of the HEMT structures explored is depicted in Figure 1, and the layer specifications applied are listed in Table 1.

**Figure 2:**
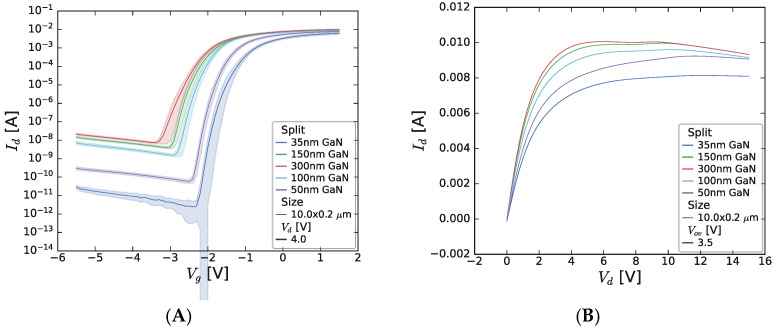
Transistor (**A**) I_d_-V_g_ and (**B**) I_d_-V_d_ characteristics measured on devices of drawn size 10 × 0.2 μm for a range of GaN thickness samples. In the case of the I_d_-V_g_, the drain bias was 100 mV, while the V_d_ was 4 V for the I_d_-V_d_ curves.

The transistor transfer and output characteristics are displayed in Figure 2 for the different GaN channel samples explored in this work. Clearly, the GaN channel thickness impacts the device threshold voltage and drain current and off-state leakage, as has previously been discussed [15] but are included here for reference.

**Figure 3:**
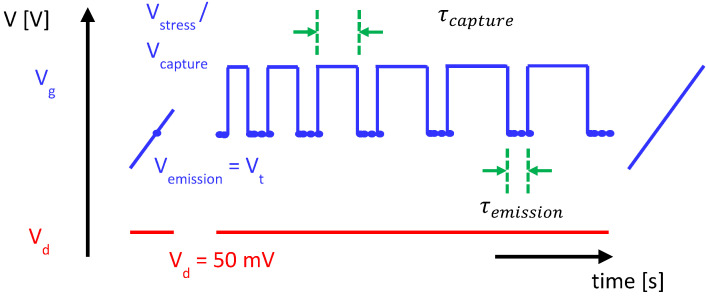
Measure–stress–measure sequence applied in this study, incorporating I_d_-V_g_ measurements in the linear regime after the final sequence.

The methodology of the charge-trapping measurements applied was as follows: the transfer characteristics of a reference device were measured to read the threshold voltage, and current at threshold condition, under a drain bias of 50 mV. Moving to the test device, the initial I_d_-V_g_ characteristics were recorded, with a current compliance incorporated, defined as double the drain current at the reference threshold current value, to ensure that the device was not stressed during the initial measurement stage. The evolution of the drain current was monitored intermittently during an applied stress voltage, with stress phases ranging in duration from ~30 ms to 2 ks. After each stress phase, the time evolution of the drain current was measured, also from ms to ks range and, using the initial IV characteristics, transformed to a threshold voltage shift (with the implicit assumption that the subthreshold characteristics during and after stress remained unchanged from the initial state, which is an assumption that is shown to be valid later in this work). This is depicted in Figure 3. Experiments were performed to monitor charge trapping as a function of gate stress bias polarity, gate bias magnitude, and measurement temperature. All devices measured had a gate width of 10 μm and a gate length of ~130 nm.

## 3. Results

### 3.1. Positive Bias Temperature Instability Results

#### 3.1.1. Charge Trapping

**Figure 4:**
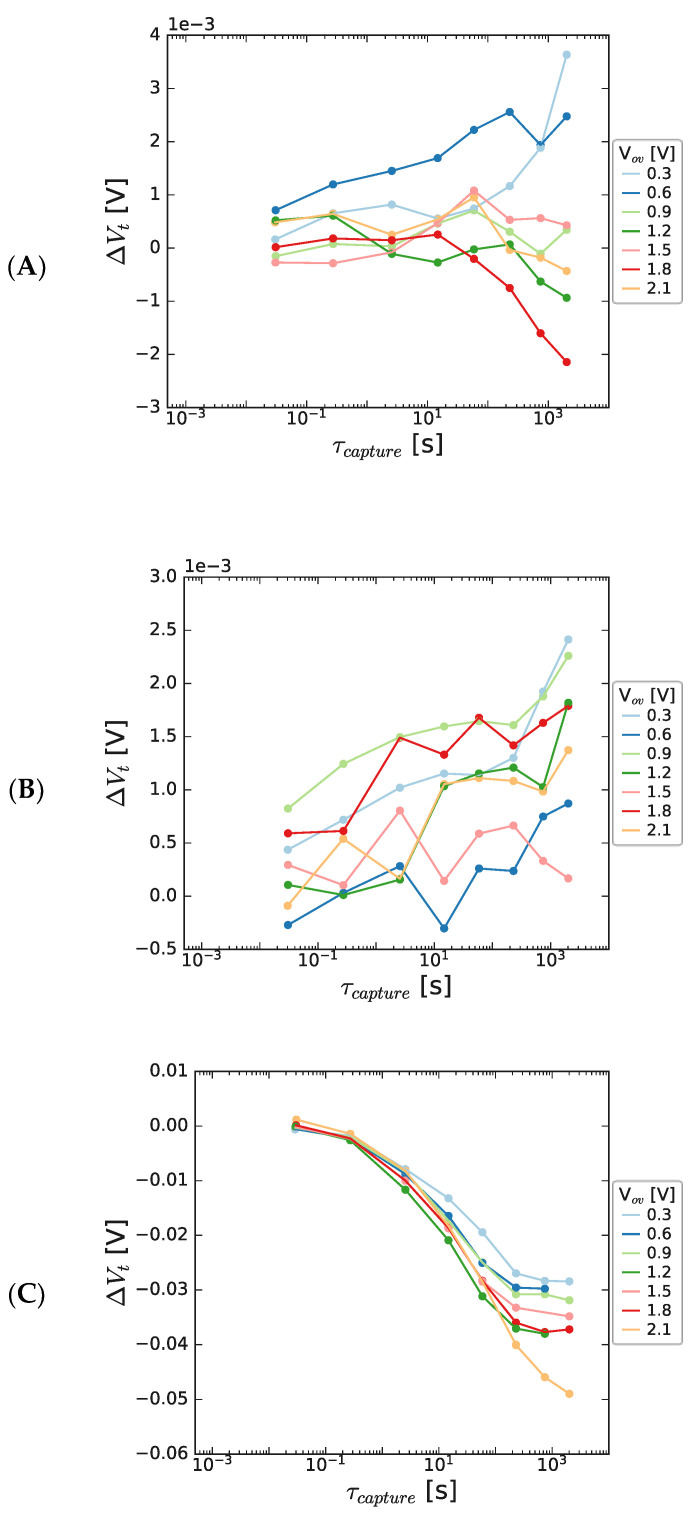
Threshold voltage shift as a function of PBTI stress time for a range of overdrive voltage values for HEMT structures, measured at 125 °C, with (**A**) 300 nm, (**B**) 150 nm, and (**C**) 50 nm GaN channel thicknesses. V_t_ shift collected 10 ms after end of each stress cycle. The stack contained 8 nm AlGaN/1 nm AlN/GaN/1 μm C-GaN.

After stressing at positive bias, the threshold voltage was monitored for devices with different GaN channel thickness values (300 nm and 150 nm), and the results are shown in Figure 4. The stress voltage was varied to explore the bias dependance of charge capture and emission. As seen in Figure 4, there are negligible threshold voltage shifts for thick channel devices, but on reducing the channel thickness to 35 nm, negative threshold voltage shifts are seen, which initially display negligible bias dependance and a monotonically reducing maximum V_t_ reduction with stress bias after longer stress times. The time dependance is broadly similar in the pre-saturation phase.

**Figure 5:**
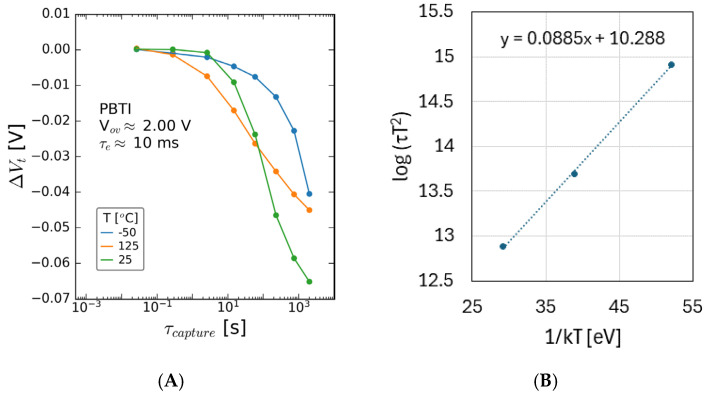
Threshold voltage shift as a function of stress time, for multiple temperatures, measured for samples with (**A**) 35 nm GaN channel and (**B**) Arrhenius plot for activation energy extraction of negative V_t_ mechanism, showing ~90 meV E_a_ for the negative V_t_ shifting mechanism. The stack contained 8 nm AlGaN/1 nm AlN/35 nm GaN/1 μm C-GaN.

The temperature dependance of the PBTI was explored for thin GaN channel samples, and the results are shown in Figure 5. As can be seen, a negative V_t_ shift of 40–70 mV is seen after ~2000 s stress, with a shorter time constant for the onset of the V_t_-reducing mechanism on increasing temperature. An Arrhenius plot of the time constant of the negative V_t_ mechanism is shown in (B) and yields an activation energy of ~90 meV.

**Figure 6:**
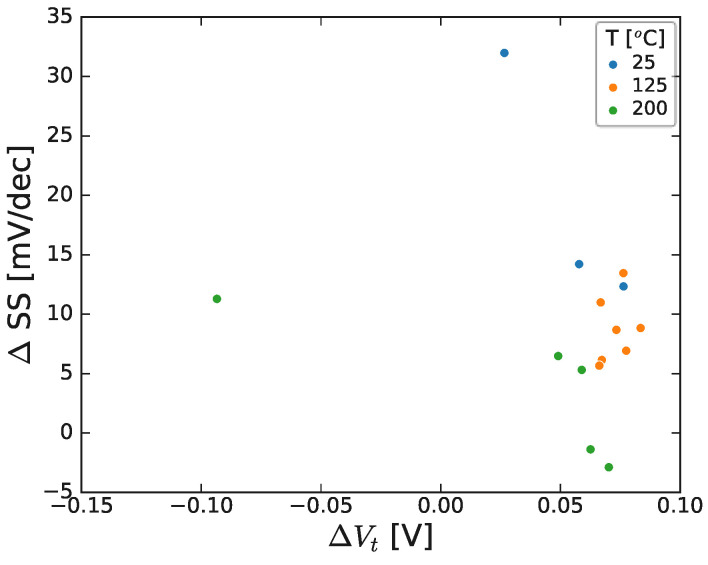
Correlation between subthreshold slope and threshold voltage shift for a range of devices, stressed at different stress conditions, and temperatures. Results shown for 300 nm GaN channel. Note each point represents a single device, stressed at a specific stress condition for ~2000 s, and subsequently had ~1000 s relaxation prior to final IV measurement. The stack contained 8 nm AlGaN/1 nm AlN/300 nm GaN/1 μm C-GaN.

The subthreshold characteristics were studied for these structures, to explore the possible link between the AlN/GaN channel surface as the possible origin of the defects responsible for the observed instabilities, and are summarized in Figure 6. There was no clear correlation between subthreshold slope and threshold voltage shift (measured from the final I_d_-V_g_ curve, i.e., after 2000 s stress and 1000 s relaxation). Similar results were obtained for the other samples, and the results suggest that the measured instabilities are not related to the 2DEG/GaN surface.

#### 3.1.2. Emission Characteristics after PBTI

**Figure 7:**
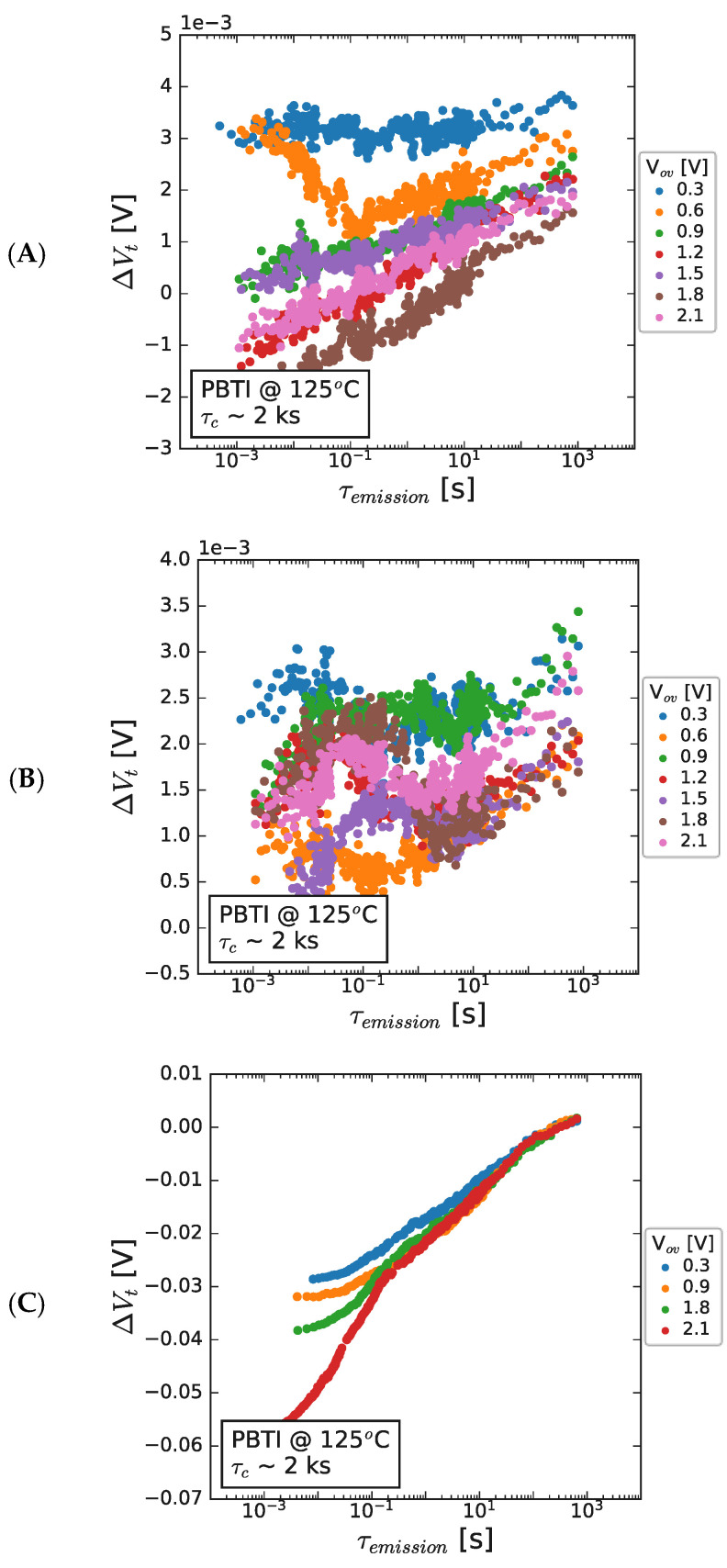
Threshold voltage shift as a function of recovery time after PBTI stressing for a range of overdrive voltage values for HEMT structures at 125 °C, with (**A**) 300 nm (**B**) 150 nm, and (**C**) 50 nm GaN channel thickness. The stack contained 8 nm AlGaN/1 nm AlN/GaN/1 μm C-GaN.

After the stress phase, the stability of the drain current was monitored (and any changes were transformed into ∆V_t_), by biasing the device at the initial V_t._ The reduction in the bias condition can enable emission from defects charged during the preceding stress phase and the results are shown for different GaN channel thickness samples in Figure 7. It is shown that, while there is a slightly negative V_t_ shift immediately after the stress phase, the subsequent biasing at the initial V_t_ results in an increase in the V_t_ value to marginally above zero in all cases. The bias dependance of recovery is not particularly strong (<4 mV), with a slightly higher (negative) V_t_ shift resulting from the higher bias conditions. It is notable that the magnitude of the V_t_ shift is significantly more negative for the 35 nm channel sample (Figure 7C), where the initial negative shift exceeds 60 mV before reducing toward and above zero during the emission phase, clearly demonstrating a strong impact of channel thickness on this negative V_t_ shift.

**Figure 8:**
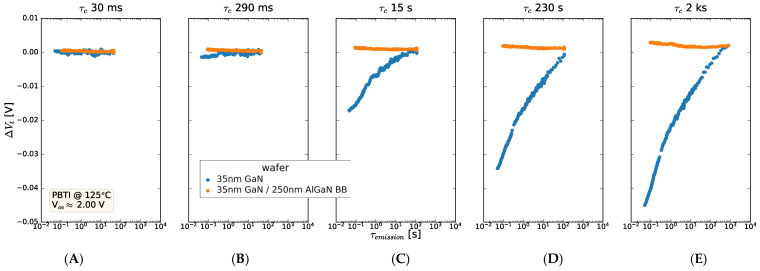
Threshold voltage shift as a function of emission time after stressing at times ranging from (**A**) ~30 ms, (**B**) 280 ms, (**C**) 15 s, and (**D**) 230 s to (**E**) 2000 s, where the stress was applied at 2.0 V overdrive. The stacks contained 8 nm AlGaN/1 nm AlN/35 nm GaN/with or without AlGaN back barrier/1 μm C-GaN.

The impact of inserting an AlGaN back barrier between the C-GaN layer and the GaN channel on the emission behavior is summarized in Figure 8, where the stress time increases from 30 ms to 2000 s on each panel. A negative V_t_ shift is seen for the 35 nm GaN sample, with C-GaN back barrier, which is not the case when an AlGaN back barrier is inserted between the GaN and C-GaN layer It is noted that the final V_t_ shift (i.e., post-emission) is similar in both cases, despite the significant capture and emission process in the C-GaN back-barrier sample. The learning from this is contribution of the C-GaN back barrier to the negative V_t_ shift and rapid emission thereafter, and the possibility to screen this effect on insertion of an AlGaN back barrier. 

To summarize the PBTI results on HEMT structures, we see a negative V_t_ shift during stress, which shows limited bias dependance. The negative V_t_ shift is enhanced by reducing the channel thickness. During the post-stress emission stage, we see an increasing V_t_ shift recovering the stress-induced shift, such that a very low (positive) V_t_ shift remains after the relaxation stage. This relaxation is also enhanced by reducing the channel thickness and is not related to changes in the interface between the GaN channel and the AlN. The effect can be allieviated for thin channel devices using an AlGaN back barrier.

### 3.2. Negative Bias Temperature Instability Results

#### 3.2.1. Charge Capture during NBTI

**Figure 9:**
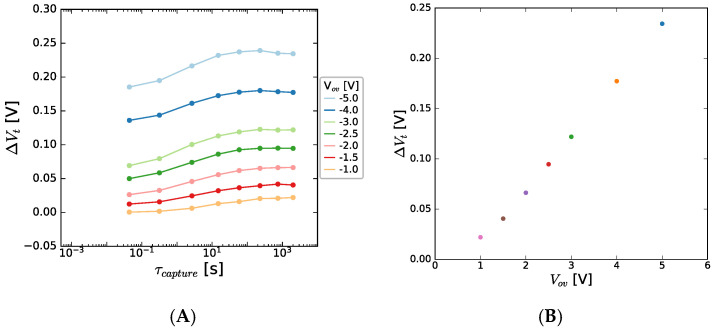
Threshold voltage shift seen as a function of (**A**) NBTI stress time for 150 nm GaN channel HEMT. A positive V_t_ shift is seen in (**B**) which increases linearly with overdrive voltage. The stack contained 8 nm AlGaN/1 nm AlN/150 nm GaN/1 μm C-GaN.

On stressing at negative voltages, *i.e.* in the OFF-state condition, the V_t_ shifts observed in a 150 nm GaN channel sample are summarized in Figure 9.

**Figure 10:**
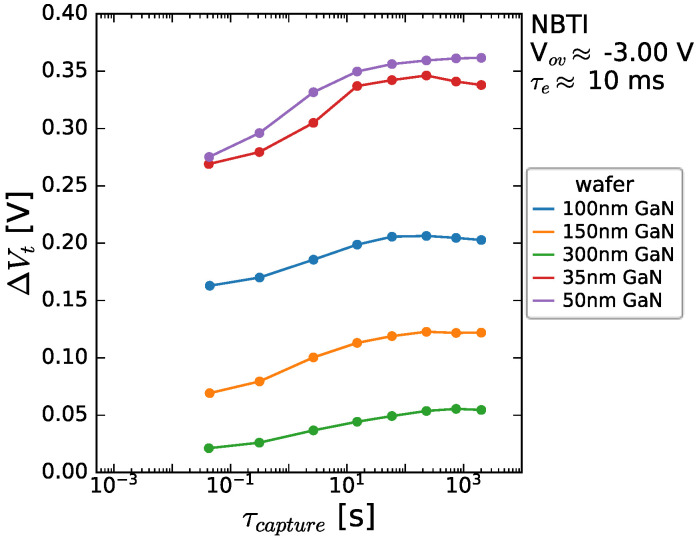
Threshold voltage shift seen as a function of stress time for a range of channel thicknesses measured during stressing at V_ov_ = −3 V at 125 °C. The stack contained 8 nm AlGaN/1 nm AlN/GaN/1 μm C-GaN.

There is a strong positive V_t_ shift seen, which increases with (negative) stress bias. This charge trapping occurs very quickly in the case of high bias, suggesting the trapping in readily accessed defect sites, and when plotted versus the overdrive voltage, a linear trend is seen. In MOSFET BTI structures, this V_t_ shift follows a power-law relationship with V_ov_, and the power-law exponent is correlated to the accessibility of the responsible defects. For Si/high-κ MOSFETs, power-law exponents of three are commonly seen for NBTI. In this case, a power-law exponent of one (i.e., a linear) correlation is seen, suggesting a similarity of energetic alignment between the Fermi level in the 2DEG and the responsible defect level, and thus a defect level that is readily accessible for capture (and emission, as shown later). Given that, during the NBTI, the transistor is biased in the OFF-state, i.e., the 2DEG is switched off, the possible impact of the buffer/back barrier are not screened. In a bid to understand further, the impact of GaN channel thickness is explored and shown to be very pronounced in Figure 10, with an enhanced V_t_ shift for the thin channel devices seen.

**Figure 11:**
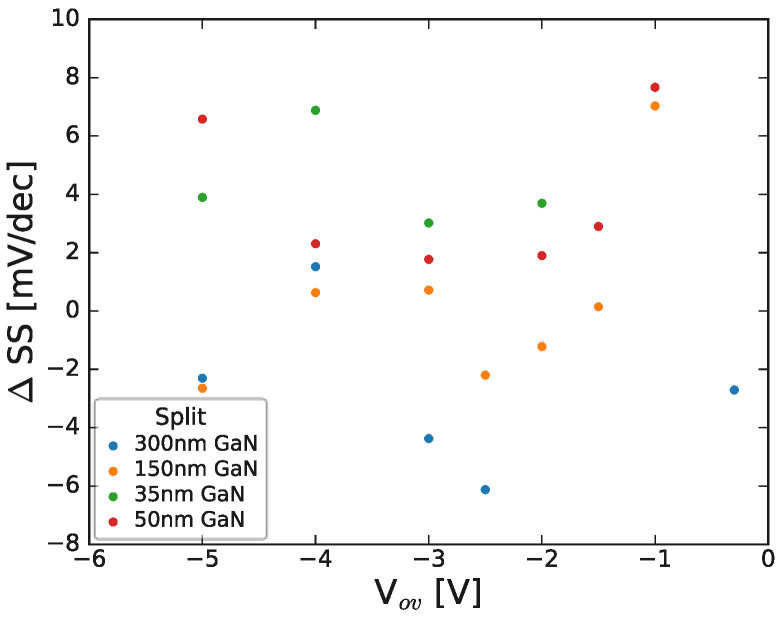
Change in subthreshold slope as a function of threshold NBTI stress (overdrive) voltage for a range of HEMTs with varying GaN channel thickness. Note that each point represents a single device stressed at a specific stress condition for ~2000 s and, subsequently, had ~1000 s relaxation prior to final IV measurement (see next section). The stack contained 8 nm AlGaN/1 nm AlN/GaN/1 μm C-GaN.

It is also noted that, given the degree of variability in the displayed dataset, there is no clear correlation between stress-induced subthreshold slope change and the applied stress bias condition, as demonstrated in Figure 11 and shown after NBTI stress at a range of bias conditions.

#### 3.2.2. Post-NBTI Emission

**Figure 12:**
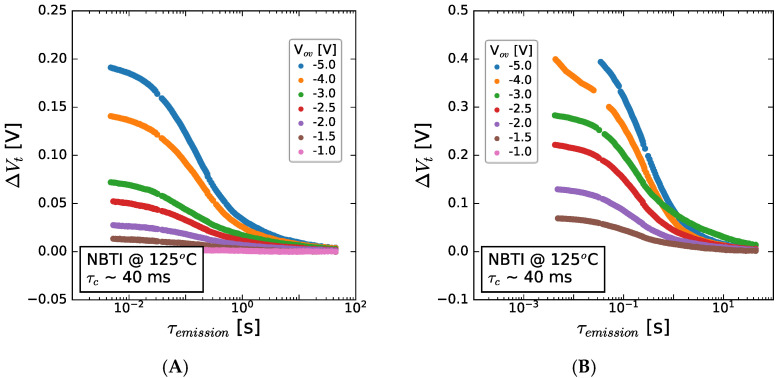
V_t_ shift seen after NBTI stress at 125 °C at a range of stress bias conditions and as a function of emission time for (**A**) 150 nm GaN channel and (**B**) 50 nm GaN channel thickness, with C-GaN back barrier present under the channel in both cases. The stacks contained 8 nm AlGaN/1 nm AlN/GaN/1 μm C-GaN.

The emission kinetics after NBTI are shown for 50 and 150 nm GaN channel devices in Figure 12. The higher V_t_ shifts described in the last section are again present and, in both cases, followed by emission, which begins at ~10 ms and before 1 s has fully emitted the trapped charge. While the V_t_ shift increases with stress bias, the emission kinetics are independent of stress bias, suggesting the same origin for the responsible defect across all bias conditions and channel thicknesses.

**Figure 13:**
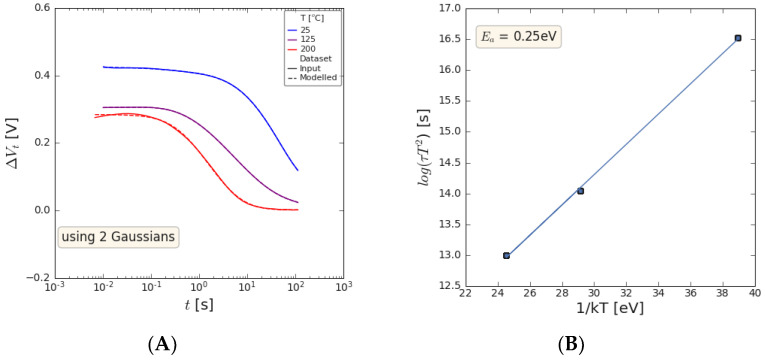
V_t_ shift seen as a function of (**A**) emission time for a range of measurement temperatures stressed at V_ov_ = −3 V and (**B**) Arrhenius plot of time constant for emission behavior shown in (**A**). Data reported for sample containing 8 nm AlGaN/1 nm AlN/35 nm GaN/1 μm C-GaN.

The temperature dependance of the post-NBTI emission is shown in Figure 13 for the 35 nm GaN channel sample, and while the magnitude of the V_t_ shift reduces with the temperature, the time constant for the emission process shows strong temperature dependance. This time constant for emission was extracted by the approach detailed in [16]. An Arrhenius plot yields an activation energy of 0.25 eV for the responsible defect.

**Figure 14:**
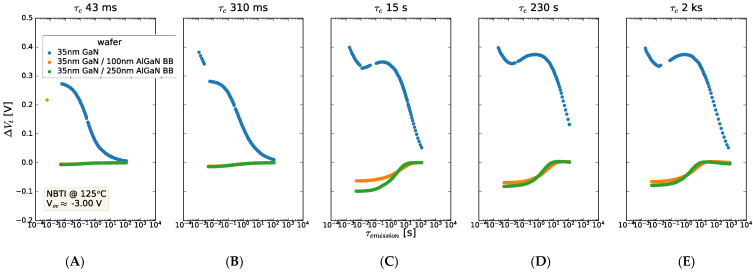
Threshold voltage shift as a function of emission time after stressing at times ranging from (**A**) ~30 ms, (**B**) 280 ms, (**C**) 15 s, and (**D**) 230 s to (**E**) 2000 s, where the stress was applied at −2.0 V overdrive. A positive V_t_ shift is seen for the 35 nm GaN sample and emission with a characteristic time constant, whereas when an AlGaN back barrier is inserted between the GaN and C-GaN layer, the positive V_t_ shift is not seen. The stack contained 8 nm AlGaN/1 nm AlN/GaN/0–250 nm AlGaN back barrier/1 μm C-GaN.

The impact of inserting an AlGaN back barrier between the GaN channel and the C-GaN back barrier is summarized in Figure 14, where the V_t_ shifts versus emission time are shown for a range of stress times (on each panel). It is clear that the rapid ∆V_t_ increase seen in the 35 nm GaN sample is not seen when an AlGaN back barrier is inserted between the 35 nm GaN channel and the C-GaN. Instead, the AlGaN back-barrier samples display a slightly negative ∆V_t_ shift, which increases towards and above zero during emission. It is also noted that the trend of the V_t_ shift seen in these AlGaN back-barrier samples is significantly different from that seen with a 150 nm GaN channel (Figure 12A). This clearly shows the impact of AlGaN screening the effect of this V_t_ shift, which is effective for the very significant shifts seen in the thin GaN channel devices.

To summarize the NBTI results, positive V_t_ shifts are seen due to negative bias stress, with increasing magnitude with (negative) bias and downscaling the channel thickness. These V_t_ shifts are recovered during the subsequent emission phase and show no correlation with the GaN/AlGaN interface characteristics. The time constant for the emission process shows temperature dependance and can be described by the Arrhenius relationship, with an activation energy of 0.25 eV. The positive threshold voltage shift can be effectively screened by the insertion of an AlGaN back barrier, with no positive V_t_ shift seen with 100 nm AlGaN between the C-GaN back barrier and the 35 nm GaN channel.

## 4. Discussion

The previous sections have presented data collected after stressing Schottky gate HEMT structures, under positive and negative bias, as a function of stress time, stress bias, temperature, and GaN channel thickness. It was shown that a negative V_t_ shift was seen after PBTI, whereas a positive shift was seen after NBTI, both of which were anomalous when compared to the MOSFET gate dielectric reliability observations. The V_t_ shifts reduced during a subsequent post-stress emission stage, (at V_t_ for both polarities). There were negligible changes in the subthreshold characteristics for both stress polarities, suggesting that defect creation at the GaN/AlN interface is not related, and the relaxation of the V_t_ shift suggests that these processes are a capture/emission related process, from pre-existing defects in the stack.

**Figure 15:**
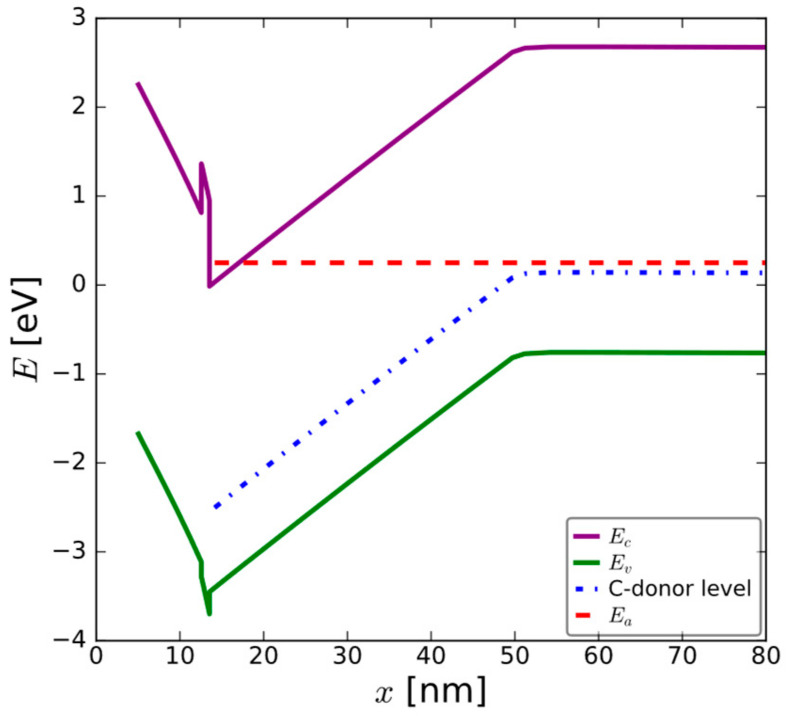
Calculated energy-band diagram for 35 nm GaN channel biased at the threshold condition. Note the energetic alignment between the C-acceptor level in the C-GaN back barrier and the Fermi level. Simulated stack contained 8 nm AlGaN/1 nm AlN/35 nm GaN/1 μm C-GaN.

To help understand the bias distribution across the constituent layers in these complex stacks, the band diagram was simulated and is shown in Figure 15. In this case, for the 35 nm GaN channel, the underlying C-GaN back barrier is also included in the figure. The role of this C-GaN back barrier needs to be considered. The widely reported C-acceptor level at E_v_ + 0.9 eV [8] becomes relevant for these structures and at the considered biasing conditions. The blue dotted lines show the energetic alignment of the C-acceptor level in the unintentionally doped GaN and predominantly C-GaN layers, while the red line shows the energetic alignment of the Fermi level at 0.25 eV above E_c min_ GaN, using the activation energy value calculated from Figure 13B. The close energetic alignment of the deep levels in the C-GaN and the 2DEG is clearly seen and can explain the V_t_ shift seen during the emission phase after NBTI, whereas trapping from the gate, directly in the C-GaN defect levels, is believed to be responsible for the positive V_t_ shifts seen in these GaN HEMT structures.

During the stress phase, as mentioned previously, for a device biased in the OFF state, there is no 2DEG, so it cannot contribute to any charge trapping. Given that increased charging is observed for a thinner GaN, it is, therefore, believed that this charging results from electron injection directly from the gate electrode to the C-GaN back barrier layer, which can subsequently be emitted to the channel on reducing the bias from the stress voltage to the emission voltage, (or initial V_t_), as depicted in Figure 14. The negative V_t_ shifts due to PBTI, resultfrom electrons being collected in the 2DEG from the C-GaN levels, which are located at higher levels energetically under these bias conditions. That the shifts are lower in the case of PBTI than NBTI is believed to be related to the lower electrical communication between the C-GaN levels and the 2DEG when the device is switched ON.

The V_t_ instabilities in these Schottky gate structures are believed to be related to the carbon-doping levels in the C-GaN back barrier. Efforts to place this layer further from the channel by increasing the channel thickness are seen to limit the charge trapping for BTI stressing at either positive or negative polarities (Figure 4 and Figure 10, respectively), albeit at a cost of a lower threshold voltage. Insertion of an AlGaN back barrier is shown to reduce NBTI (significantly) and PBTI (significantly) with the compromise of increased thermal resistance to dissipate the generated heat during operation [7].

## 5. Conclusions

This study reports on experiments performed on the bias temperature instability of Schottky gate HEMT structures for applications as power amplifiers in RF/mm wave communication systems. It is shown that the threshold voltage shifts during bias temperature instability measurements, with positive V_t_ shifts during NBTI and negative V_t_ shifts during PBTI. Both the positive and negative shifts reduce during a post-stress subsequent emission step. Neither the positive nor the negative V_t_ shifts are correlated with changes in the GaN/AlN interface characteristics, leading to the conclusion that the cause is capture and emission in pre-existing defect levels within the AlGaN/AlN/GaN/C-GaN stacks. The energy-band diagram suggests direct access between the Fermi level in the 2DEG at V_t_ and the dopant level in the C-doped GaN back barrier. The bias induced band diagram modulation and enabled access to and from this defect level for both biasing conditions.

## Figures and Tables

**Table 1:** Process splits of GaN HEMT samples explored in this study.

Back Barrier	GaN Channel	AlN	AlGaN
1 μm C-GaN	300 nm	1 nm	15 nm→8 nm
1 μm C-GaN	150 nm	1 nm	15 nm→8 nm
1 μm C-GaN	100 nm	1 nm	15 nm→8 nm
1 μm C-GaN	50 nm	1 nm	15 nm→8 nm
1 μm C-GaN	35 nm	1 nm	15 nm→8 nm
1 μm C-GaN and100 nm AlGaN	35 nm	1 nm	15 nm→8 nm
1 μm C-GaN and250 nm AlGaN	35 nm	1 nm	15 nm→8 nm

## Data Availability

The data presented in this study may be available on request from the corresponding author.
